# The Role of Nuclear Factor Kappa B (NF-κB) in the Immune Response against Parasites

**DOI:** 10.3390/pathogens11030310

**Published:** 2022-03-02

**Authors:** Piotr Bąska, Luke J. Norbury

**Affiliations:** 1 Division of Pharmacology and Toxicology, Department of Preclinical Sciences, Institute of Veterinary Medicine, Warsaw University of Life Sciences-SGGW, 02-786 Warsaw, Poland; 2 Department of Biosciences and Food Technology, School of Science, STEM College, RMIT University, Bundoora, VIC 3083, Australia; luke.norbury2@rmit.edu.au

**Keywords:** NF-κB, parasites, immune response, p65, p50, RelA

## Abstract

The immune system consists of various cells, organs, and processes that interact in a sophisticated manner to defend against pathogens. Upon initial exposure to an invader, nonspecific mechanisms are raised through the activation of macrophages, monocytes, basophils, mast cells, eosinophils, innate lymphoid cells, or natural killer cells. During the course of an infection, more specific responses develop (adaptive immune responses) whose hallmarks include the expansion of B and T cells that specifically recognize foreign antigens. Cell to cell communication takes place through physical interactions as well as through the release of mediators (cytokines, chemokines) that modify cell activity and control and regulate the immune response. One regulator of cell states is the transcription factor Nuclear Factor kappa B (NF-κB) which mediates responses to various stimuli and is involved in a variety of processes (cell cycle, development, apoptosis, carcinogenesis, innate and adaptive immune responses). It consists of two protein classes with NF-κB1 (p105/50) and NF-κB2 (p100/52) belonging to class I, and RelA (p65), RelB and c-Rel belonging to class II. The active transcription factor consists of a dimer, usually comprised of both class I and class II proteins conjugated to Inhibitor of κB (IκB). Through various stimuli, IκB is phosphorylated and detached, allowing dimer migration to the nucleus and binding of DNA. NF-κB is crucial in regulating the immune response and maintaining a balance between suppression, effective response, and immunopathologies. Parasites are a diverse group of organisms comprised of three major groups: protozoa, helminths, and ectoparasites. Each group induces distinct effector immune mechanisms and is susceptible to different types of immune responses (Th_1_, Th_2_, Th_17_). This review describes the role of NF-κB and its activity during parasite infections and its contribution to inducing protective responses or immunopathologies.

## 1. Introduction

Parasites are a diverse group of organisms comprised of three major groups: protozoa, helminths, and ectoparasites. During the course of their evolution, they have gained the ability to live in or on hosts and gather food at the hosts’ expense. They may feed on both humans and animals; up to 2.0 billion [1] people may suffer from parasitic infections, and losses in animal production are estimated at many US$ billions per year [2]. It is not in the interest of the parasite to kill the host, consequently chronic, long-lasting infections can be common. 

The persistency of many infections occurs in part due to the immunomodulatory abilities of the parasites [3]. During the thousands of years of co-evolution with host organisms, parasites have learned to trick, evade and suppress the host immune system. Parasites’ mechanisms of immunomodulation are very effective and diverse, and this has earned them the nickname “masters of regulation” [4]. Parasites can cleave antibodies [5,6,7], induce apoptosis in macrophages [8] and eosinophils [9], interfere with cytokine signaling networks [10,11,12] and cytokine release [13,14,15,16], and induce regulatory B cells [17]. However, exploring the diversity and range of parasite immunomodulation is outside the scope of this article; this topic has been reviewed elsewhere [18,19,20,21,22,23].

NF-κB plays a critical role in mediating responses to a remarkable diversity of external stimuli, and thus is a pivotal element in multiple physiological and pathological processes and is a powerful orchestrator of the immune response [24,25,26]. There are many reports in the literature regarding the role of NF-κB during specific parasite infections. The goal of this article is to explore parasites’ interplay with NF-κB. In this manuscript, we review the existing knowledge regarding NF-κB expression and activity during infections with *Plasmodium* spp., *Trypanosoma* spp., *Leishmania* spp., *Toxoplasma* spp., cestodes, nematodes, and flukes in the context of the parasite life cycle, occupied niche, and impact on the immune response. By bringing these disparate reports together and reviewing the topic in one publication we hope to shed new light on this issue, highlighting the role NF-κB plays during parasite infections and why it represents an important target for parasite manipulation. 

## 2. Structure and Function of NF-κB

NF-κB is a transcription factor; a family of five proteins can dimerize to form NF-κB complexes: NF-κB1 (p105), NF-κB2 (p100), RelA (p65), RelB and c-Rel [27]. NF-κB1 and NF-κB2 undergo processing into mature forms, p50 and p52, respectively. All the NF-κB proteins share the Rel homology domain (RHD)—which allows them to form homo- or heterodimers [27] and bind DNA [28]—additionally p65, RelB and c-Rel contain TAD (transcriptional activation domain), enabling activation of gene expression [28]. A number of heterodimers may be formed, but the most commonly present in most cell types is p50/p65 [29], while c-Rel containing dimers are constrained predominately to hematopoietic origin cells [30]. 

Prior to activation, latent NF-κB dimers are coupled to inhibitors (IκB) which sequester the transcription factor in the cytoplasm. In most cells, NF-κB is associated with IκBα, IκBβ or IκBε; additionally, p105 and p100 contain an inhibitor within their sequence, thus also functioning as inhibitors (while p50 and p52 homo- and hetero-dimers repress NF-κB-dependent transcription) [31]. Translocation of NF-κB to the nucleus and activation requires dissociation from the inhibitor, which takes place upon phosphorylation of the inhibitor by an IκB kinase (IKK). In the canonical activation pathway, IKK is composed of catalytically active IKKα, IKKβ, and the regulatory subunit IKKγ (NEMO) [31], and its activity promotes p50, RelA, and c-Rel activation through IκB phosphorylation [32]. In the non-canonical pathway, NIK (Nuclear Factor κB-inducing Kinase) [33] activates IKKα which phosphorylates the p100 subunit of p100/RelB leading to migration of p52/RelB to the nucleus [32]. Both pathways regulate the expression of a distinct and overlapping set of genes [34], regulating innate and adaptive immune responses [27]. 

NF-κB dimers bind to sequences known as κB sites with the general consensus GGGRNWYYCC (R–purine, W–adenine or thymine, Y–pyrimidine, N–any base); however, different dimers may show differing specificity for target sequences [35]. Moreover, each particular NF-κB protein may undergo a variety of post-translational modifications [36] which impacts their stability, degradation, affinity to binding sites, and interactions within the dimer and with other transcription factors [37]. 

## 3. Role of NF-κB in the Immune Response

**Figure 1:**
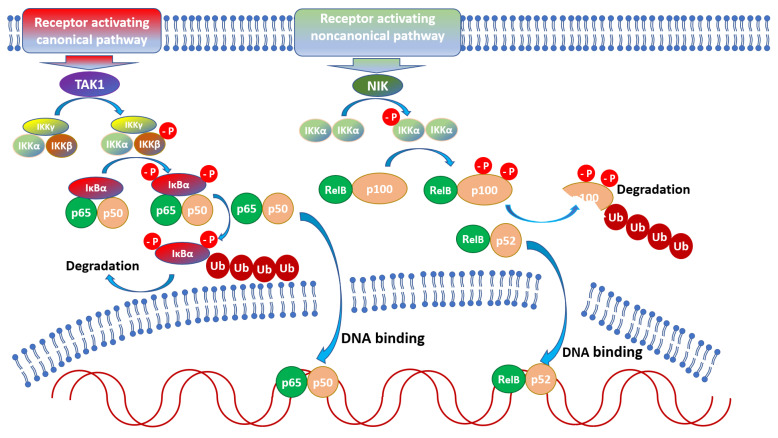
Schematic model of NF-κB activation through canonical and non-canonical pathways. Canonical activation involves TGF-β activated kinase-1 (TAK1) which phosphorylates Inhibitory Kappa B Kinase β (IKKβ) complexed with IKKα and IKKγ (NEMO). This leads to phosphorylation of the α Inhibitor of κB (IκBα), its detachment from the p56/p50 dimer, ubiquitination, and proteasomal degradation. Released p65/p50 dimer migrates to the nucleus and binds to DNA sequences leading to transcription of appropriate genes. During the noncanonical pathway, NF-κB-inducing kinase (NIK) phosphorylates the IKKα dimer which phosphorylates p100 leading to its disruption and release of the RelB/p52 dimer. The dimer migrates to the nucleus and regulates the transcription of particular genes.

NF-κB is crucial for appropriate immune system functioning at all stages, from the development of primary and secondary lymphoid tissues, through hematopoiesis, to recognizing Danger-Associated Molecular Patterns (DAMPs) or Pathogen-Associated Molecular Patterns (PAMPs) and regulating effector mechanisms of immune cells [38,39]. There are two main pathways of NF-κB activation: canonical and noncanonical [40] (Figure 1). 

**Figure 2:**
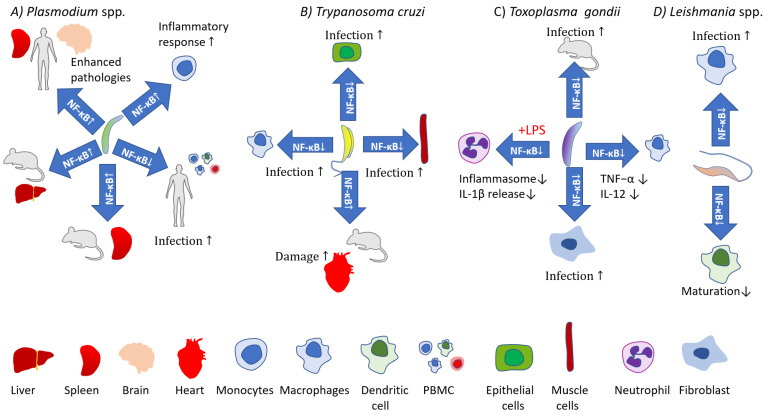
The impact of *Plasmodium* spp., *Trypanosoma cruzi, Toxoplasma gondii,* and *Leishmania* spp. on NF-κB activity and outcomes. (**A**) *Plasmodium* spp. increase NF-κB activity in specific cell populations which is associated with pathology in the brain, inducing cerebral malaria symptoms (apoptosis in brain endothelial cells and intravascular leukocytes), and may facilitate hidden parasite populations in the spleen. *Plasmodium* spp. also trigger an inflammatory response in monocytes, but patients with decreased NF-κB activity in PBMCs show more severe malaria symptoms. (**B**) Reduced NF-κB activity facilitates infection of *T. cruzi*. Enhanced NF-κB activity in heart tissue during *T. cruzi* infections leads to heart failure. (**C**) RelB-deprived mice do not survive *T. gondii* infection. *T. gondii* deactivate NF-κB signaling, reducing the immune response in macrophages and neutrophils. (**D**) *Leishmania* spp. reduce NF-κB activity in infected macrophages and DC, facilitating parasite survival.

**Figure 3:**
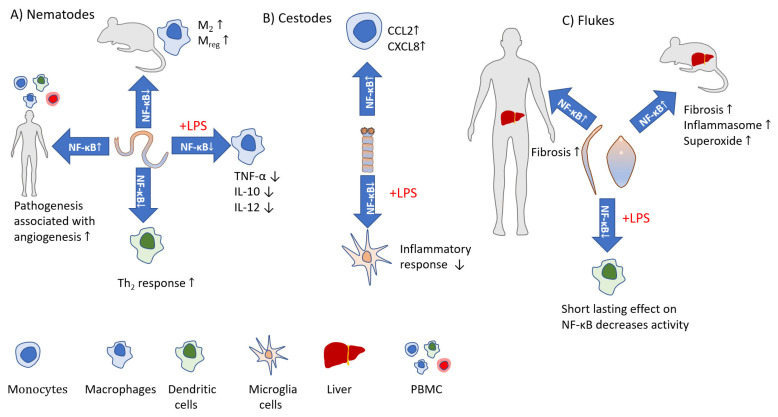
The impact of helminths on NF-κB activity and outcomes. (**A**) *B. malayi* infection results in decreased NF-κB activity and induces M_2_ and eventually M_reg_ macrophages, while patients with lymphatic pathology show increased angiogenesis associated with NF-κB activation. *H. polygyrus* induces semi-maturation of DCs and induces Th_2_ and regulatory events through modulation of NF-κB activity. Products released by *T. spiralis* affect NF-κB activity in LPS-activated macrophages, significantly reducing proinflammatory cytokine production. (**B**) *T. solium* larval antigens activate the NF-κB pathway in monocytes inducing chemokine release. *M. corti* antigens inhibit LPS-induced inflammatory phenotypes in microglia cells via NF-κB modulation. (**C**) *F. hepatica* tegumental antigens temporarily prevent LPS-induced NF-κB in DC, suppressing maturation. *S. mansoni* induces NF-κB activation in human hepatic stellate cells which is associated with liver fibrosis; a similar situation occurs in *S. Japonicum-*infected mice.

Activation of the canonical pathway is mediated through TAK1 and has rapid and transient effects, whereas the noncanonical pathway is slow, persistent, and with the hallmark of NIK activation [32]. Moreover, the canonical pathway is activated by various stimuli including PAMPs, DAMPs, and numerous receptors, while the noncanonical pathways are induced by a specific set of receptors, e.g., B-cell-activating factor belonging to TNF family receptor (BAFFR), lymphotoxin β-receptor (LTβR), receptor activator for nuclear factor κB (RANK), TNFR2, Fn14, CD30, and CD27 [41]. Both pathways are indispensable for the appropriate regulation of the balance between immune tolerance and inflammation. Due to the number of potential NF-κB dimers [42], and interactions with other factors [43] the role of NF-κB during the course of infections is very complicated. Both positive and negative implications during parasite infections are reviewed below and depicted in Figure 2 and Figure 3.

## 4. *Plasmodium* spp.

Malaria is a disease caused by *Plasmodium* spp. Over 40% of the human population is estimated to be at risk of infection [44]. A vast majority of cases occur in Africa, and despite a global trend towards reductions in the number of infections over recent decades, over 241 million malaria cases and 627,000 deaths were estimated to have occurred globally in 2020 [45]. Whether host responses contribute to pathology or protection can be hard to determine, and while proinflammatory responses to malaria infections are critical to controlling the disease, excessive inflammation is linked to pathology (Table 1). NF-κB is a highly important orchestrator of the immune response during *Plasmodium* infections [46,47,48]. In general, during an infection NF-κB is increased and it has pivotal and specific roles in pathogenesis and the immune response, being implicated in several processes. Cerebral malaria is considered the most severe clinical complication of infection and proinflammatory cytokines, cytoadherence, and endothelial activation are considered to have roles in pathogenesis [49]. Exposure to *P. falciparum*-infected erythrocytes has been shown to induce nuclear translocation of NF-κB p65 in human brain microvascular endothelial cells in vitro, along with upregulation of the NF-κB activation cascade, of NF-κB subunits (p100, p105, c-REL, RELB) and NF-κB inhibitory proteins (IκBα, IκBε)—this results in increased proinflammatory chemokine/cytokine release (CCL20 and TNF-α) and intercellular adhesion molecule 1 (ICAM-1) surface expression [48,50]. ICAM-1 is linked with the adhesion of infected erythrocytes to epithelial cells and the associated disease pathology. While this can occur in the brain, it can also occur in other organs. Upon exposure to extracellular vesicles containing *P. vivax* proteins, human spleen fibroblasts show upregulation of ICAM-1, linked to nuclear translocation of NF-κB [51]. This has been postulated to facilitate the formation of hidden parasite populations in the spleen relatively safe from control measures. Patients with cerebral malaria also show increased p65 translocation to the nucleus in neurons, glial cells, epithelial cells, and leukocytes [46]. These changes correlate with histopathological changes in the brain, with NF-κB p65 modulating apoptosis in brain endothelial cells and intravascular leukocytes during cerebral malaria [46]. NF-κB has also been shown to regulate apoptosis in Kupffer cells and lymphocytes in the liver during severe *P. falciparum* infection [52]. Other cell types also show upregulation of NF-κB pathways during infection. Increased translocation of p65 and p50 to the nucleus and degradation of IκBα occurs in monocytes following exposure to trophozoites or hemozoin. This coincides with enhanced activity of monocyte matrix metalloproteinase-9 and increased proinflammatory cytokine production, effects also important to malaria pathogenesis [53]. Interestingly, it has been reported that while significantly elevated active p65 levels occur in peripheral blood mononuclear cells (PBMCs) of *P. vivax* patients and *P. falciparum* patients with mild symptoms, those with severe *P. falciparum* malaria only showed increased levels of active p65 after treatment, with NF-κB expression being negatively correlated with IL-10 levels [54]. During infections, the malaria parasite can induce upregulation of NF-κB pathways in a variety of cell types. The full role of NF-κB during malaria infection is dependent on cell type and likely influenced by parasite strain and the genetics of the host. While further study is needed to fully elucidate NF-κB’s roles, it is evident that during severe infections there is a link between NF-κB upregulation and several effects that play a role in malaria pathogenesis.

## 5. *Trypanosoma* spp.

The *Trypanosoma* genus is comprised of several species, but only *Trypanosoma cruzi* and *T. brucei* (*T. b. gambiense and T. b. rhodesiense*) cause disease in humans, Chagas disease and sleeping sickness, respectively. Other species cause disease in animals and may also be models for human infection. Both Chagas disease and sleeping sickness are significant problems in sub-Saharan and tropical climates. Trypomastigotes can infect a range of cell types (Table 2). Several trypanosome species have been shown to activate NF-κB in epithelial cells, endothelial cells, and fibroblasts [55,56]. This involves a rapid increase in p65 and p65 translocation to the nucleus, as well as phosphorylation of IKKα/β, resulting in induction of a pro-inflammatory response through NF-κB -dependent expression of TNF-α, IL-1β, and IL-6 cytokines, nitric oxide, and adhesion molecules (E-selectin, VCAM-1, and ICAM-1) [55,56,57,58] Activation of NF-κB is a determinator of *T. cruzi* intracellular survival and tissue specificity. The ability for *T. cruzi* to induce NF-κB activation is highly dependent on cell type and appears to be inversely correlated with a cell’s susceptibility to infection. The high susceptibility of muscle cells to infection by *T. cruzi* has been proposed to be in part due to a failure to induce NF-κB activation [55]. Activation of endothelial cells via the NF-κB pathway occurs with intact trypomastigotes, however, trypanosome trans-sialidases have also been shown to mediate this activity via α-2,3 sialylated receptors [56]. Toll-like receptors (TLRs), likely through recognition of parasite glycoisnositolphospholipids and glycosylphosphatidyl inositol, have also been shown to be involved in *T. cruzi*-mediated increased NF-κB and the resultant pro-inflammatory response [57,59,60]. Other receptors that recognize *T. cruzi* and are involved in subsequent activation of NF-κB have been identified; macrophage galactose-C type lectin (MGL1) receptor is critical for the optimal activation of macrophages during *T. cruzi* infection. MGL1 is proposed to recognize soluble *T. cruzi* Lysate Antigen (TcAg) [61]. A trypanosome antigen that can disrupt the protective host response via modulation of NF-κB is cruzain from *T. cruzi*. Cruzain cleaves p65, this prevents macrophage activation during early infection, facilitating parasite survival and the spread of infection [62]. For efficient parasite removal, the immune system must maintain a balance between effective Th_1_ and Th_reg_ immune responses. A Th_1_ response, mediated mainly by TNF-α may be efficient at clearing the parasite, but without appropriate regulating mechanisms (mainly through IL-10) exacerbated inflammation may lead to increased pathology and disease severity. This is evident in mice with impaired IL-10 production, which are less resistant to *T. congolense* and *T. brucei* infection and show higher degrees of inflammation and immunopathologies due to dysregulated immunoregulation [63]. A major orchestrator of this regulation is NF-κB, with p65/p50 and p50/p50 complexes important players in regulating pro- and anti-inflammatory pathways, respectively [64]. p50 plays a role in down-regulation of the inflammatory response [65], and p50^−/−^ mice fail to maintain an appropriate balance between Th_reg_/Th_1_, leading to increased liver injury during *T. congolense* infection, associated with TNF-α overproduction [66]. While induction of NF-κB pathways to promote proinflammatory responses can confer protection against trypanosomes as outlined above, a prolonged response can confer pathogenicity. *T. cruzi*-induced proinflammatory responses in human colonic epithelial cells have been linked to Megacolon, a major pathology of Chagas disease [57]. Additionally, activation of NF-κB -dependent inflammatory responses in cardiomyocytes, and vascular endothelial cells by *T. cruzi* may contribute to vascular dysfunction and injury, and chronic cardiomyopathy [58,67,68].

## 6. *Toxoplasma* spp.

*Toxoplasma gondii* is an obligate intracellular Apicomplexan parasite able to inhabit a broad spectrum of mammals [69]. It has been estimated that up to one-third of the world’s population may be seropositive to this parasite [70]. The host–pathogen interplay and molecular basis of host resistance are complicated, but a pattern showing a key role for a balance between IL-1β, TNF-α, IL-12, and IFN-γ exists [71,72,73,74,75]. Upon disruption of this balance the protective but over-expressed Th_1_ cytokines (IFN-γ, IL-1β, TNF-α, and IL-12) lead to immunopathologies [76]. NF-κB is involved in the positive and negative regulation of these cytokines [77,78,79] and is a crucial factor required for eliciting a protective response (Table 3). This is demonstrated by the fact that RelB^−/−^ mice develop impaired innate and adaptive immune responses and do not survive *T. gondii* infection [80]. *T. gondii* infection in mice results in a global increase in NF-κB activity [81]. However, most data indicate *T. gondii* induces a suppressing NF-κB phenotype in infected macrophages in mice leading to delayed IL-12 production and failure to produce TNF-α. Despite increased p65 phosphorylation [82], and IκBα phosphorylation [83], translocation of p65 and c-Rel is not observed [81,83,84]. This process of NF-κB suppression requires active invasion by the parasite [84], and recently downregulation of miR-187 by the parasite has been implicated in p65 phosphorylation and mediation of IL-12 expression [82]. On the other hand, infected human monocytes show an activated phenotype with an increase in both p65 phosphorylation [85] and p65 translocation [74]. This leads to IL-1β induction [86], and also requires active invasion by the parasite, with dense granule protein GRA15 playing a role, and parasite strain lineage also important [74]. LPS-induced IL-1β levels also appear unaffected in *T. gondii* infected monocytes [86]. 

Of course, it is worth remembering that the use of different models may produce different results. A well-established model to investigate the immune response is based on simultaneous stimulation of cells with lipopolysaccharide (LPS) and the factor under investigation, to observe if there is an abrogation of the LPS-induced proinflammatory response [93,94]. Conflicting results have been reported for mouse macrophages, some indicating *T. gondii* has the ability to abolish LPS-induced p65 translocation [83], whereas others indicate LPS-induced p65 translocation is unaffected, as is LPS-induced nuclear NF-κB-binding activity [92]. Nevertheless, the consensus is that *T. gondii* infection interferes with NF-κB activation at a step downstream of these processes and effectively dampens the host immune response, facilitating parasite survival and multiplication [82,83,84,92]. Another LPS-activated immune cell population—neutrophils—also skew their phenotype towards suppression when infected with *T. gondii*; dampened inflammasome activity and IL-1β release is associated with reduced p65 (Ser536) phosphorylation and inhibition of IκBα degradation–classical hallmarks of reduced NF-κB activity [86]. Data from non-immune cells also confirms *T. gondii*’s abilities to deactivate NF-κB. Despite human fibroblasts showing some signs of increased NF-κB activity upon *T. gondii* exposure, like IKK-dependent degradation of IκB, the phenotype is actually one of reduced transcription due to altered downstream processes: reduced phosphorylation of p65 and no p65, p50, or c-Rel nucleus accumulation [89]. Phosphorylation of p65 at Ser468 by virulence factor ROP18, targeting it for degradation, and blocking nuclear translocation has also been demonstrated as a means to inhibiting the host NF-κB pathway in fibroblasts and macrophages [91]. These results highlight the importance of phosphorylation as a means by which the parasite can mediate NF-κB pathway inhibition [37]. 

This contrasts with a report of mouse fibroblasts which show a more activated phenotype, with induced p50 and p65 translocation to the nucleus, enhanced DNA binding by p50, p65, RelB, and p52, and phosphorylation (though no observed degradation) of IκB upon *T. gondii* infection [90]. This response was reported to result in an anti-apoptotic phenotype. NF-κB is considered an anti-apoptotic factor, therefore, despite efforts by the parasite to dampen NF-κB activity complete abrogation of activity is not likely to be in the interest of the parasite; apoptosis would destroy the niche it lives in–the cell [95]. Some *Toxoplasma*-infected cells appear resistant to apoptosis [96] and show NF-κB dependent anti-apoptotic gene expression patterns [90]; moreover, NF-κB deprived mice (p65^−/−^) are unable to prevent apoptosis hallmarks in infected cells [97]. While our understanding of the interplay between this parasite and NF-κB is still in its infancy, the findings presented here indicate *T. gondii* has developed sophisticated mechanisms to manipulate host NF-κB to effectively modulate the host response to facilitate parasite survival. The dampening of host responses is also likely to minimize immune-mediated host pathology.

## 7. *Leishmania* spp.

*Leishmania* is a protozoan parasite that inhabits phagolysosomes [98]. Data shows that 12 million people worldwide suffer from leishmaniasis [99]. Clinical manifestations of the disease depend on the *Leishmania* species and are classified as: cutaneous, visceral, mucocutaneous, and diffuse cutaneous leishmaniasis [100]. *Leishmania* resides in host macrophages, as well as neutrophils and inflammatory monocytes [99]. Like other protozoan parasites, *Leishmania* has developed mechanisms to survive in the host and evade the immune response. A coordinated immune response (Table 4) is required to eliminate *Leishmania*, and effective parasite removal is associated with proinflammatory IL-12, TNF-α, and IFN-γ, whereas IL-4, IL-10, IL-13, and TGF-β are associated with parasite survival [101]. The key cytokine believed to be involved in parasite survival is IL-10, with IL-10 knockout (KO) mice resistant to infection [102,103]. It is therefore not surprising that the parasites’ efforts at dampening the proinflammatory response, through increased IL-10 release are intense. The pivotal role of NF-κB in immune response regulation, including as a positive and negative regulator of macrophage gene expression [104], also key to *Leishmania* invasion, means NF-κB offers an attractive target for the parasite to manipulate. Upon contact with *L. major* amastigote*,* human monocytes preferentially induce p50/p50 and p50/c-Rel, with inhibition of p50/p65 translocation [105]. p50/p50 complexes are associated with IL-10 [64], and infected monocytes release increased concentrations of both IL-10 and TNF-α [105]. While IL-10 is expected to be beneficial for the parasite and is implicated in *Leishmania* survival, TNF-α is involved in inflammation and is putatively protective. Interestingly, infection of macrophages by *L. major* promastigotes results in inhibition of IL-12, a response beneficial to the parasite, though this is not due to inhibition of NF-κB activation [106]. 

*Leishmania* parasites may modulate the NF-κB pathway in numerous ways and for various reasons. Upon infection of human or mouse macrophages, the promastigote stage of several *Leishmania* species (but not *L. aethiopica* or *L. tarentolae*) can cause cleavage of p65 into p35. This is dependent on *Leishmania* protease gp63. p35 is involved in the expression of specific chemokines and the parasite has been proposed to induce those favorable to its survival [98,109]. Cleavage of p65 to p35 has also been reported following dendritic cell infection by *L. infantum* promastigotes [111]. *L. aethiopica* amastigotes have been shown to manipulate a variety of signaling pathways, including NF-κB, in human macrophages during spreading [109]. This results in reduced p65 expression and phosphorylation (Ser32/36) of IκB, indicating downregulation of NF-κB. This down-regulation of NF-κB leading to cell apoptosis is expected to facilitate the spread of the parasite [109]. Conversely, decreased expression of IκB was also reported which is expected to induce p65 translocation and NF-κB pathway activation and may indicate pleiotropic impacts of *Leishmania* on NF-κB pathways. 

*Leishmania* species can promote silent infections under certain conditions; this can sometimes be achieved through the modulation of NF-κB pathways. It has been observed that when *L. donovani* promastigotes infect mouse macrophages, there is no change in the expression of cytosolic or nuclear p50 or p65 expression in response. This results from active downregulation of NF-κB, mediated through Hypoxia Inducible Factor-1α (HIF-1α) and miR-210 [110]. This leads to decreased pro-inflammatory cytokine expression and NO production by the macrophages and facilitates parasite survival. The generation of a permissive environment is also evident upon *L. amazonensis* amastigote infection of mouse dendritic cells. The infection leads to pleiotropic inhibition of TLR/NF-κB/NLRP3 pathways, promoting transcriptional activation of the alternative NF-κB pathway which is proposed to lead to MHC class I-restricted antigen presentation and stalling of dendritic cell maturation [107]. Stalled mouse dendritic cell maturation is also evident upon infection by *L. infantum* promastigotes [111]. Here the parasite impairs NF-κB through cleavage of p65, although no impact on IkBα expression was reported.

It is difficult to draw one clear conclusion regarding the processes and roles of NF-κB modulation during *Leishmania* infection based on the varied data in the literature. Moreover, there may not even be one clear conclusion. Authors have analyzed various laboratory models, cell types, and different *Leishmania* species, hence contrasting results may be expected. This is supported by the results of Nogueira et al. who evaluated responses to various *Leishmania* species (*L. braziliensis*, *L. infantum*, *L. amazonensis*) and showed that only *L. amazonensis* was able to induce p65 translocation to the nucleus and a pro-inflammatory response in a particular laboratory model [108]. The variability among species to cleave p65 to p35 should also be noted [98,109]. Species-specific and stage-specific variations, as well as the development of effective immune responses complicate the situation; nevertheless, from the known data emerges a pattern which generally indicates that *Leishmania* modulates the NF-κB pathway towards a dampening of TNF-α expression and production of an environment that facilitates parasite survival.

## 8. Helminths

Helminths are a divergent group of multicellular parasites (Cestoda, Nematoda, Trematoda). Due to their size, like protozoan parasites, they cannot be neutralized by phagocytosis; moreover, their abilities to trick and evade the host immune system allow them to efficiently regulate the immune response towards a Th_2_ type. This is beneficial for the parasite and also arguably the host—the parasite survives and gains reproductive success while the host does not suffer from immunopathologies. The ability to dampen Th_1_ responses has led to an exploration of the use of helminths (or their products) as treatments for allergies and autoimmune diseases (as reviewed elsewhere [112,113]). The high prevalence of helminth infections has also led to research regarding the development of vaccines. These have led to numerous studies regarding vaccine trials, the impacts of helminth antigens on symptoms of autoimmune diseases (or allergies), and cytokine patterns during infections, yet only a small proportion of the research has focused on characterizing the intracellular mechanisms of the immune response in the context of NF-κB and this topic seems to have been relatively neglected to date.

### 8.1. Cestodes

Helminth parasites excrete and secrete a range of molecules during host invasion. These molecules function at the host-parasite interface and are often how parasites modulate the host immune response (Table 5). The excretory-secretory product (ES) from *Taenia crassiceps* is known to skew immune responses through the downregulation of proinflammatory pathways. One way *T. crassiceps* can achieve this is through modulation of dendritic cells, which play a major role in the initiation of Th_2_ polarization [114]. *T. crassiceps* ES can modulate signaling in dendritic cells, including in the NF-κB pathway, leading to significantly attenuated LPS-induced p65 phosphorylation. This contributes to the blocking of dendritic cell maturation and a dampening of proinflammatory IL-12 and TNF-α, which affects eventual T-cell responses [114]. *Mesocestoides corti* soluble antigens also exhibit NF-κB-modulated immunosuppressive properties. *M. corti* helminth soluble factors (HSFs) inhibit LPS-induced p65 phosphorylation and acetylation in microglia leading to abrogation of IL-6 and TNF-α release [115]. This parasite suppression of host inflammation and immunity provides another example of how parasites may actively modulate host responses to produce asymptomatic phases of the disease. Parasite somatic antigens function to maintain the parasite’s metabolism, physiological processes and regulate homeostasis, and generally have less contact with the host immune system. While parasites actively suppress host immune responses, parasite damage or death can sometimes lead to an inflammatory response and pathology. This may occur during *Taenia solium* infections. *T. solium* larval somatic antigens activate the NF-κB pathway in human monocytes, through increased IkB-α degradation and enhanced DNA binding by p65, p50, or c-Rel. This results in a release of the chemokines CXCL8 and CCL2, which may have a role in immune cell recruitment, which may be a factor associated with inflammation and pathology [116]. 

### 8.2. Nematodes

Insights into the role of the NF-κB pathway during nematode infections come from the well-established model organism *Trichuris muris*. A detailed and nuanced role for NF-κB in mounting a protective response has been revealed during in vivo experiments (Table 6). Mice able to clear *T. muris* infections show enhanced NF-κB activity with elevated levels of IL-4 and IL-13 and lower levels of IFN-γ, demonstrating a protective Th_2_ response and skewing away from a susceptible Th_1_ response [118]. c-Rel KO mice maintain resistance to infection, whereas p105/50 (NF-κB1) and p100/52(NF-κB2) KO mice develop chronic infection, with the highest immunopathologies observed among p105/50 deficient mice, correlating with elevated IFN-γ concentrations [118]. p50/p50 complexes are associated with regulatory IL-10 [64,65,119] suggesting a role for IL-10 in controlling intestinal inflammation. The data from KO studies indicate roles for components of the NF-κB pathway and demonstrates NF-κB utilization during helminth infections as an inductor of a protective regulatory response. Colonic epithelium cells show increased p65 phosphorylation upon exposure to *T. muris* ES which leads to the release of proinflammatory factors [120]. 

During *T. spiralis* infection, NF-κB (though P2X7R-mediated NLRP3 activation) modulates the killing capacity of macrophages, with NF-κB downregulation resulting in decreased killing capacity [123]. *T. spiralis* ES from various life stages can reduce LPS-induced p65 expression and nuclear translocation [124,125]. The resulting inhibition of LPS-induced pro-inflammatory (TNF-α, IL-1β, IL-6, IL-12) cytokine expression, and induction of regulatory cytokines (IL-10, TGF-β) indicates a role during infections in the modulation of macrophage responses towards phenotypes conducive to worm survival and host health [124,125]. 

Modulation of macrophage regulation is also observed during *Brugia malayi* infections. Downregulation and impaired activation of NF-κB-p65 and NF-κB-p50/105 in mouse macrophages during the course of infection contributes to macrophage polarization towards M_2_ and M_reg_ phenotypes and downregulation of proinflammatory IL-12 release and increased secretion of Th_2_/Th_reg_ cytokines: IL-4 and IL-10, respectively [126]. IL-10 and IL-12 release is also impaired in human DCs exposed to *B. malayi* microfilariae [130]; however, pathologies during infection are associated with increased Th_1_/Th_17_ responses [131] and enhanced angiogenesis [132]. NF-κB is involved in both angiogenesis [133] and inflammation [134] regulation. Although the basic levels of angiogenic factors do not differ between asymptomatic and filarial lymphedema (lymphatic pathology) patients, the cells from lymphedema patients produce higher levels of vascular endothelial growth factor (VEGF)-C [127] and NF-κB activation has a role in elevated angiogenic growth factor production, and associated pathology, in these patients [127]. While several nematode derived molecules may contribute to the development of angiogenesis, Jothi et al. identified a prominent angiogenic factor from *B. malayi:* Asparaginyl–tRNA Synthetase [135], which acts through the NF-κB pathway [121]. 

Prevention of DC maturation, upon incubation with L4 larvae of *Heligmosomoides polygyrus* [128] and the ability of adult stage *H. polygyrus* somatic antigens to inhibit proliferation and apoptosis of host immune cells [129] are other examples where parasite regulation of the NF-κB pathway is utilized by nematodes to modulate host immune responses and facilitate survival. 

### 8.3. Flukes

The formation of liver granuloma and fibrosis following egg deposition is a serious pathology that can occur during schistosomiasis. The TNF-α level, raised by parasite egg antigens, is positively correlated with parasite burden [136], and increased TNF-α mediates increased morbidity [137]. As the NF-κB pathway is involved in TNF-α expression, this indicates increased NF-κB activity. While no data are available from immune cells (Table 7), liver and colonic cells show increased p65 expression, translocation to the nucleus, and phosphorylation (Ser276) [136,138,139], with involvement of NF-κB in apoptosis. The role of NF-κB in pathology is supported by the fact that its inhibition in vivo prevents granuloma and fibrosis [140,141]. *Fasciola hepatica* uses a variety of molecules to modulate host immune responses to facilitate its survival and manage host pathology, driving anti-inflammatory responses and Th_1_ suppression; it is considered a potent immune modulator. Some, but not all of the immune-modulatory effects induced by the parasite involve interaction with the NF-κB pathway. *F. hepatica* tegumental antigens (Teg) prevent NF-κB activation (p65 expression) in dendritic cells. This leads to suppression of dendritic cell maturation and function—suppressing induction of proinflammatory cytokines and cell surface markers [142]. The ability of *F. hepatica* to modulate responses [93,143,144] has led to attempts to use its components to treat a range of diseases and conditions. An example is the use of *F. hepatica* extracellular vesicles (EV) to treat colitis. EV were shown to suppress induced p65 translocation in a mice colitis model, effectively reducing the release of proinflammatory cytokines TNF-α, IL-6, and IL-17A, and attenuating clinical symptoms of colitis [145]. *F. hepatica* EV, Teg, and ES are comprised of a variety of molecules and their action is a sum of the action of all the antigens present. Therefore, these antigens may impact NF-κB in various ways. Two ES components, glutathione S-transferases (GST) and fatty acid binding protein (FABP) have separately been shown to be able to block LPS-induced NF-κB-dependent gene expression [146,147,148]. These molecules can suppress macrophage LPS-induced pro-inflammatory cytokines, activate a suppressive dendritic cell phenotype, and induce anti-inflammatory effects that attenuate septic shock and promote survival in a mouse model [146,147,148].

## 9. Conclusions

The NF-κB pathway is vital in regulating immune function and it plays an important role during infections by parasites, being integral to the formation of protective responses, or indeed pathologies. For example, NF-κB is pivotal to the induction of protective proinflammatory responses against trypanosomes and to the activation of macrophages during *Leishmania* or *T. cruzi* infections. Alternatively, hosts deficient in NF-κB pathway elements or unable to appropriately activate or regulate NF-κB pathways are often more susceptible to infections or pathologies. This has been demonstrated with *T. gondii* [80], *T. muris* [118], and trypanosome infections [63], while the high susceptibility of muscle cells to *T. cruzi* infection is proposed to be linked to a failure to induce NF-κB activation [55]. Altered NF-κB activation in certain cell types during parasite infections can also lead to increased pathology, as observed in the brain in cases of cerebral malaria [32]. 

The wide impact of NF-κB on various processes and in numerous cell types and its importance to mounting effective immune responses means that modulation of NF-κB pathways represents a vital strategy for many parasites to employ to facilitate survival. As such, parasites have evolved numerous strategies to either up- or down-regulate NF-κB. These include, but are not limited to, direct phosphorylation of NF-κB pathway proteins or their inhibitors [91], binding of certain receptors to activate specific pathways [56,57,61], or cleavage of NF-κB proteins via parasite proteases [62,98,111]. While each modulates NF-κB activity, the resulting outcomes of these actions are dependent on the cell types involved and whether NF-κB activity is increased or decreased.

The data presented in this review demonstrates that NF-κB has many crucial roles, including many essential to mounting an effective response against protozoan and metazoan parasites. By presenting several relevant host-parasite interactions involving parasite modulation of the NF-κB pathway we have highlighted how it is a target for parasites to create an environment within the host conducive to survival. We hope this has shed new light on this important regulator of immunity for the reader.

## Figures and Tables

**Table 1:** *Plasmodium* spp. impact on NF-κB activity and outcomes.

Parasite	Infection/Life Stage/Antigen	Host/Location/Model	Effect on NF-κB	Outcome
*P. falciparum* [53]	Trophozoites Haemozoin	Human monocytes	Translocation of p65, p50 to nucleus of monocytes; degradation of IκBα.	Enhanced activity of MMP-9, enhanced production of proinflammatory cytokines TNF and IL-1β.
*P. falciparum P. vivax* [54]	Infected human patients	Peripheral blood mononuclear cells	Elevated phospho-NF-κB p65 levels (*P. vivax* and uncomplicated *P. falciparum* infection). Reduced phospho-NF-κB p65 levels in complicated *P. falciparum*, but elevated post-treatment.	Possible association of lower NF-κB features in patients with complicated *P. falciparum* malaria may be induced by increased IL-10 level during the course of the infection.
*P. falciparum* [46]	Fatal cases of human cerebral malaria	Brain tissue	Increase in expression of nuclear NF-κB p65 in the brain (neurons, glial cells, ECs, and intravascular leukocytes).	NF-κB p65 levels correlate with histopathological changes (apoptosis) in brain. NF-κB p65 modulates apoptosis in the brain endothelial cells and intravascular leukocytes (but not glial cells, neurons) of fatal cerebral malaria patients.
*P. vivax* [51]	Plasma-derived extracellular vesicles from *P. vivax* patients	Human spleen fibroblasts	Translocation of NF-κB to the nucleus.	Upregulation of ICAM-1 surface expression, facilitating adhesion of infected reticulocytes.
*P. falciparum* [48]	Trophozoite-stage *P. falciparum* infected erythrocytes	Human brain microvascular endothelial cells	Upregulation of NF-κB activation cascade, upregulation of NF-κB subunits (p100, p105, cREL, RELB) and upregulation of NF-κB inhibitory proteins (IκBα, IκBε). Increased p65 translocation to nucleus.	Increased proinflammatory cytokine release (CCL20 and TNF-α).
*P. falciparum* [52]	Infected patients	Liver tissue	p65 expression in B cells and Kupffer cells correlates with severity of the disease.	Apoptosis of Kupffer cells and portal tract lymphocytes is related to NF-κB activation.
*P. falciparum* [50]	Trophozoite-stage *P. falciparum* infected erythrocyte	Human brain microvascular endothelial cells	Induction of nuclear translocation of NF-κB (p65).	Increase in ICAM-1 surface expression.

**Table 2:** *Trypanosoma* spp. impact on NF-κB activity and outcomes.

Parasite	Infection/Life Stage/Antigen	Host/Location/Model	Effect on NF-κB	Outcome
*T. cruzi* [57]	Trypomastigotes	Primary human colon epithelial cells	Increase in p65 and IKKα/β phosphorylation, increase in phosphorylation of NF-κB upstream regulators (TAK1 and IRAK4).	Modulated expression of NF-κB signaling molecules, proposed to promote pro-inflammatory signaling pathways.
*T. cruzi* [61]	Soluble *T. cruzi* lysate antigen	Mouse macrophages	Increased phosporylation of NF-κB p65 (partially dependent on MGL1 receptor).	IL-10, TNF-α, and nitric oxide (NO) production.
*T. cruzi* [68]	Experimental infection	Thoracic aortic rings from infected C57BL/6 mice	Increase in expression of NF-κB p65.	Increased expression of COX-2 and thromboxane synthase in aortas leading to vascular contraction.
*T. congolense*,*T. vivax*, *T. b. gambiense*, *T. b. brucei* [56]	Mouse infection with tripomastigotes, Sialidases Trans-sialidases	Bovine aortic endothelial cells, in vivo experiments 13 different human and murine endothelial cell lines	Translocation of NF-κB to nucleus, IκBα phosphorylation, NF-κB activation via classical pathway (mediated by parasite trans-sialidase).	Endothelial cell activation, and subsequent pro-inflammatory response–production of cytokines IL-1β and IL-6, nitric oxide and expression of adhesion molecules.
*T. cruzi* [67]	Trypomastigotes	Human cardiomyocytes (AC16)	Increased nuclear translocation of p65, enhanced expression of NF-κB dependent genes.	Enhanced mRNA expression of TNF-α and IL-β.
*T. cruzi* [55]	Trypomastigotes	Mink lung epithelial cells (Mv1lu), murine endothelial line (SVEC4–10), primary cultures of human fibroblasts, rat myoblasts (L6E9 and H9c2), primary human vascular smooth muscle cells, bovine aortal muscle cells	Nuclear translocation of p65 in epithelial cells, endothelial cells, and fibroblasts, enhanced expression of NF-κB dependent genes.	NF-κB activation by parasites limits infection levels whereas experimental blocking of NF-κB signaling increases parasite burden.
*T. cruzi* [58]	Trypomastigotes	Human umbilical vein endothelial cells (HUVEC)	Nuclear translocation of active NF-κB (p65 and p50).	Induction of vascular cell adhesion molecule 1 (VCAM-1) and E-selectin and the upregulation of intercellular adhesion molecule 1 (ICAM-1).
*T. cruzi* [62]	Wild *T. cruzi* strain Cruzain-deficient *T. cruzi strain*	J774 macrophages	Proteolytic cleavage of NF-κB p65 by cruzain.	Impairment of macrophage activation pathways (reduced IL-12, increased L-arginase).

**Table 3:** *Toxoplasma gondi* impact on NF-κB activity and outcomes.

Parasite	Infection/Life Stage/Antigen	Host/Location/Model	Effect on NF-κB	Outcome
*T. gondii* [82]	Tachyzoites	Mouse primary peritoneal macrophages	Phosphorylation of p65 through downregulation of miR-187.	Delayed production of IL-12.
*T. gondii* [87]	Dense granule protein GRA16	Human Non-Small Cell Lung Cancer H1299 cells deficient in p53	GRA16 prevents NF-κB activation; decreased total and nuclear levels of p65, decreased IKKβ level, decreased phosphorylation of IkBα.	Decrease in cell survival, induce cell apoptosis.
*T. gondii* [86]	Tachyzoites	Human neutrophils	Reduction in LPS-induced IκBα degradation and p65 phosphorylation.	Reduction in release of LPS induced IL-1β.
*T. gondii* [88]	Tachyzoites of virulent & avirulent strains	In vivo: mice. In vitro: bone marrow derived macrophages, RAW 264.7, adherent peritoneal macrophages, HELA cells	Virulent strain resulted in less p65 translocation to the nucleus and IκBα phosphorylation compared to avirulent strain.	Avirulent strains induced increased TNF-α and IL-12 release compared to virulent strains. Avirulent and virulent strain polarized macrophages towards M1 and M2 phenotypes, respectively.
*T. gondii* [89]	Tachyzoites of virulent strain	Human foreskin fibroblasts, NIH 3T3 fibroblasts, MEF, HeLa, and COS cell lines, as well as primary cultures of mouse and human macrophages	Lack of nuclear p65/RelA translocation despite IκB degradation. No increase in expression of NF-κB dependent genes upon infection.	The results show that *T. gondi* may use undefined mechanisms to interfere with NF-κB signaling.
*T. gondii* [84]	Tachyzoites of virulent strain	Mouse bone marrow-derived macrophages	Inhibition of LPS-induced NF-κB translocation.	Blocked production of proinflammatory TNF-α and delayed (24 h) IL-12 in response to LPS.
*T. gondii* [81]	Tachyzoites of virulent strain	In vivo: mice. In vitro: mouse bone marrow derived macrophages	In vivo: activation of NF-κB, higher expression of p65, p50 from 24 h. In vitro: no p65 and cRel translocation to nucleus.	In vitro: reduced capacity to increase transcription of IL-12, IL-18, and iNOS in response to LPS and IFN-γ.
*T. gondii* [90]	in vitro infection	3T3 mouse embryonic fibroblasts, 3T3 p65^−^/^−^ fibroblasts	nuclear translocation of p50 and p65. Higher affinity to DNA for p50, p52, p65, and RelB. Phosphorylation but no IκB degradation.	Upregulation of antiapoptotic responses.
*T. gondii* [91]	*T. gondi ROP18^+^* strain *T. gondi ROP18^−^* strain	Human foreskin fibroblast, RAW264.7 (mouse macrophage cell line), U937 (human macrophage cell line)	ROP18^+^ strain induced p65 phosphorylation at Ser468 and promotes its degradation.	ROP18^+^: reduced LPS-induced IL-6, IL-12, and TNF-α; M2-biased phenotypes. ROP18-: enhanced LPS-induced IL-6, IL-12, and TNF-α; M1-biased activation. This strain has a relative inability to inhibit the NF-κB pathway.
*T. gondii* [92]	Tachyzoites	Mouse bone marrow-derived macrophages	No change in LPS induced p65 accumulation in nucleus as well as NF-kB binding to DNA. Significant diminished ability of p65 to bind to TNF-α promoter.	Describes *T. gondi*’s ability to interfere with TNF-α transcription.
*T. gondii* [74]	Tachyzoites of type I, II and III	Primary human monocytes and THP-1 cells	Type II: increased p65 accumulation in nucleus.	Type II: expression of IL-1β.
*T. gondii* [85]	Tachyzoites	Primary human peripheral blood monocytes	Increase in phosphorylation of p65.	Expression of IL-1β.
*T. gondii* [80]	Cysts	Wild type and RelB^−/−^ C57B6 mice.	Infection induces NF-κB DNA binding activities of p65 and RelB containing complexes in the spleen.	RelB^−/−^ mice show high mortality in response to the infection with negligible levels of IFN-γ and diminished NK cell activity.
*T. gondii* [83]	Tachyzoites	In vivo: Mice (injected intra peritoneally with tachyzoites). In vitro: mouse peritoneal macrophages macrophage cell lines (RAW 264.7 and THP-1)	In vivo: no NF-κB translocation in macrophages or neutrophils within 4h of infection. In vitro: rapid IκB phosphorylation and degradation but NF-κB p50-p65 heterodimers did not translocate to the nucleus.	In vitro: little or no production of IL-12 and TNF-α; LPS triggering unable to promote IL-12 and TNF-α production.

**Table 4:** *Leishmania* spp. impact on NF-κB activity and outcomes.

Parasite	Infection/Life Stage/Antigen	Host/Location/Model	Effect on NF-κB	Outcome
*L. major* [105]	Amastigotes	Promonocytic human cell line U937 and fresh human peripheral blood monocytes	Inhibition of DNA binding activity of p50/p65 heterodimer. Induction of p50/p50 and p50/c-Rel heterodimer.	Increase in IL-10 and TNF-α secretion.
*L. amazonensis* [107]	Amastigotes Amastigotes opsonized with antibiodies	murine immature bone marrow-derived DCs	Expression of p65 and RelB. Down regulation of genes encoding for activators (IL-1 receptors, TIRAP, MYD88, TIFA, EIF2AK2, USP7), upregulation of genes encoding inhibitors of the NF-κB pathway (OPTN, TNFAIP3, TAX1BP1, PTPN1, USP10).	Alternative NF-κB pathway is favored, likely promoting MHC I-restricted antigen presentation. Amastigote infection does not change maturation status whereas antibody opsonized amastigotes induce semi-mature DC phenotype.
*L. braziliensis*, *L. infantum* (WT and modified), *L. amazonensis* [108]	Promastigote extracellular vesicles	Murine peritoneal Macrophages, THP-1 macrophages	Only *L. amazonensis* EV were able to induce p65 translocation.	Only *L. amazonensis* EV induced NO, TNF-α, IL-6, and IL-10 via TLR4 and TLR2.
*L. aethiopica* [109]	Amastigotes	Human THP-1 cells	Reduction of total p65 protein, lower expression of total Iκ-Bα protein, decrease in IkBα phosphorylation (Ser 32/36), no p65 cleavage to p35.	NF-κB signal pathway downregulation proposed to promote induction of apoptosis to facilitate spreading.
*L. donavani* [110]	Promastigotes	Mouse macrophages and mouse macrophages with inhibited miR-210 expression	During the infection p50 and p65 expression relatively unchanged in cytoplasm and nucleus. During the infection and miR-210 inhibition increased p50/p65 translocation to nucleus.	Infection: marginally elevated TNF-α, IL-12, and IL-10. miR-210 inhibition during infection: TNF-α, IL-12 more elevated, IL-10 less elevated, increased O2- and Nox.
*L. major* [106]	Promastigotes	BM derived mouse macrophages	Infection did not inhibit NF-κB activation: no impact on p65 translocation, no impact on LPS-induced IκBα degradation, no activation of NF-κB dependent gene expression.	Infection inhibits LPS-induced IL-12 (not NF-κB related)
*L. infantum* [111]	Promastigotes	Mouse bone marrow-derived DCs	Cleavage of p65 to p35, IκB-α phosphorylation and degradation unchanged.	Slightly increased expression of IL-12, unchanged IL-10, IL-6, TNF-α expression. Reduced LPS-induced IL-12 and IL-6, increased IL-10, TNF-α unchanged.
*L. donovani*, *L. major*, *L. mexicana*, *L.* (Viannia) *braziliensis*,*L. tarentolae* [98]	Promastigotes	B10R macrophage, primary bone marrow-derived macrophages, mouse and human macrophage cell lines Raw264.7, J774 and THP-1	Excluding non-pathogenic *L. tarentolae*: p65 cleaved into p35, p35 translocation to nucleus, nuclear p35/p50.	Induction of chemokine gene expression (MIP-2/CXCL2, MCP-1/CCL2, MIP-1a/CCL3, MIP-1b/CCL4).

**Table 5:** Cestodes impact on NF-κB activity and outcomes.

Parasite	Infection/Life Stage/Antigen	Host/Location/Model	Effect on NF-κB	Outcome
*Taenia crassiceps* [114]	Product released by the parasite (*Tc*-ES)	Mouse BMDCc	*Tc*-ES did not induce p65 phosphorylation (Ser536), but attenuated LPS-induced p65 phosphorylation (Ser536).	Inhibition of DC maturation, cytokine production, and the ability of LPS-treated DCs to prime Th_1_ responses.
*Taenia solium* [116]	Larval antigen (*Ts-*Ag)	Human primary monocytes	Increased degradation of IkB-α but not IkBβ; increased DNA binding by p65, p50, and cRel.	Induced CCL2, CXCL8, and CCL3 secretion.
*Mesocestoides corti* [115]	Helminth soluble factors (HSFs)	Mouse microglia cells	Decrease in LPS-induced p65 phosphorylation and acetylation.	Inhibition of LPS-induced secretion of IL-6 and TNF-α cytokines.
*Taenia crassiceps* [117]	Fractionated *Taenia* glycans Lewis sugars	Mouse spleen cells	p65 level increased, p50 level was not affected.	Release of IFN-γ from spleen cells stimulated with N glycans upon stimulation with *Taenia* glycans; Lewis X sugars upregulated p65 expression and had no impact on p50 expression.

**Table 6:** Nematodes impact on NF-κB activity and outcomes.

Parasite	Infection/Life Stage/Antigen	Host/Location/Model	Effect on NF-κB	Outcome
*Brugia malayi* [121]	Recombinant *B. malayi* Asparaginyl-tRNA Synthetase	Colitic mice	Changes in NF-κB signaling pathway.	Mitigation of colitis symptoms.
*Wuchereria bancrofti* [122]	microfilarial sheath protein (*Wb*-MfP)	Mouse macrophages (RAW 264.7) HEK293-TLR4 cells	Increased NF-κB phosphorylation Enhanced expression of NF-κB dependent gens.	*Wb*-MfP induces proinflammatory response through TLR4.
*Trichuris muris* [120]	Product released by the parasite (*Tm*-ES)	Colon epithelial cell line (CMT93)	Dynamic increase in p65 phosphorylation. Increase at 4 h post-exposure, back to baseline level by 24 h.	Increased release of proinflammatory factors IFN-γ, TNF, and CCL2.
*Trichuris muris* [118]	Infection with *T. muris* eggs	B6 mice and NF-κB1 KO, NF-κB2 KO, c-Rel KO mice	Enhanced activity of NF-κB upon infection in B6 mice.	c-Rel KO mice: expelled worms and lymph node cells released low level of IFN-γ. NF-κB1 KO and NF-κB2 KO mice: unable to terminate infection, NF-κB1 KO mice lymph node cells released high levels of IFN-γ, and mice developed intestinal immunopathology.
*Trichinella spiralis* [123]	newborn larvae (NBL)	Human macrophage cell line (U937)	Downregulation of NF-κB following P2X7R blockade.	Inhibits NLRP3 inflammasome activation and decreases macrophage capacity to kill *T. spiralis* larvae.
*Trichinella spiralis* [124]	ES products of L1 muscle larvae	Mouse macrophages (RAW264.7)	Reduced LPS-induced p65 expression levels.	Increased regulatory IL-10, reduced LPS-induced proinflammatory (TNF-α and IL-12) and regulatory cytokines (IL-10).
*Trichinella spiralis* [125]	ES of muscle larvae (ML) and adult worms (3 and 5-day-old) and newborn larvae (NBL)	Macrophage cell line (J774A.1)	Reduced LPS-induced p65 nuclear translocation.	Increased levels of anti-inflammatory cytokines (IL-10, TGF-β) and arginase 1, and (ML) iNOS. Reduced LPS-induced expression of proinflammatory (TNF-α, IL-1β, IL-6, IL-12) cytokines and (Ad3+Ad5+NBL) iNOS.
*Brugia malayi* [126]	Experimental infection with *B. malayi* L3 larvae	BALB/c mice	Expression downregulation for p65 and p50/105 in M_2_ macrophages and dramatic decrease in p65 and p50/105 in M_reg_.	Alternatively activated (M_2_) and regulatory (M_reg_) macrophages appeared 3 and 7 dpi, respectively.
*Brugia malayi* [127]	Humans with lymphedema Humans with aspymptomatic infection	TLR ligands	Use of NF-κB inhibitor.	Diminished production of Angiopoetin-I and VEGF-A in response to TLR2 ligand in the presence of NF-κB inhibitor.
*Heligmosomoides polygyrus* [128]	L4 larvae	Immature DC line (JAWS II)	Decreased translocation of p50 NF-κB into the nucleus.	Relative lack of DC activation: TNF-α, TGF-β, IL-6, MCP-1 unchanged, decreased expression of regulatory cytokine (IL-10) and proinflammatory factor (IL-12p70), increased expression of IL-4 (Th_2_ type).
*Heligmosomoides polygyrus* [129]	Adult stage somatic antigens (*Hp*-Ag)	MLN cells from naive and infected mice	Various changes in p65 and p50 levels in cytoplasm and nucleus nucleus.	Inhibition of apoptosis.

**Table 7:** Flukes impact on NF-κB activity and the main outcomes.

Parasite	Infection/Life Stage/Antigen	Host/Location/Model	Effect on NF-κB	Outcome
*Schistosoma mansoni* [138]	Infected patients	Liver biopsy	Increased p65 presence in hepatic stellate cells’ (HSC) cytoplasm and enhanced translocation to nucleus in infected patients.	Increase in apoptotic HSC number in schistosome-induced fibrosis.
*Schistosoma mansoni* [139]	Infected mice	Mice	Increase of p65 expression in liver.	May promote inflammasome activation.
*S. mansoni* [149]	Infected mice	Mice	Increased p65 (Ser 276) phosphorylation in colonic tissue.	NF-κB shows significant role during the course of schistosomiasis.
*Fasciola hepatica* [145]	Secreted extracellular vesicles (*Fh*-EV)	Colitic mice	Diminished p65 expression in response to *Fh*-EV in colitic mice.	Protective effect of *Fh*-EV: amelioration of pathological colitis symptoms and reduced the amount of pro-inflammatory cytokines.
*Fasciola hepatica* [142]	Tegumental antigen (*Fh*-Teg)	Mouse bone marrow-derived DCs	Suppressed LPS–induced expression of p65.	*Fh*-ES suppressed LPS–induced IL-12p70, IL-10 and TNF-α release; modulated LPS-stimulated DC cytokine production and cell surface marker expression.
*Fasciola hepatica* [147]	Native *F. hepatica* glutathione S-transferase (n*Fh*-GST)	Mouse bone-marrow derived macrophages; Mice with induced septic shock, THP-1 cells	Blocked TLRs from inducing NF-κB activation.	Reduced expression of LPS induced mRNA encoding IL-1β and TNF-α and release of IL-1β, IL-12p70, IL-6, IFN-γ, TNF-α and IL-2.
*Fasciola hepatic*a [148]	*F. hepatica* fatty acid binding protein (*Fh-*12)	Mouse bone-marrow derived macrophages; Mice with induced septic shock, THP-1 cells, HEK293 cells	Blocked LPS-induced NF-κB activation.	Reduced mRNA expression encoding IL-1β, IL-12p35, IL-12p40, TNF-α, IL-6, NOS-2 Reduced release of GM-CSF, IL-12p70, IL-3, IL-9, IL-10, IL-15, TNF-α and IFN-γ.
*Fasciola hepatica* [146]	*F. hepatica* sigma class glutathione transferase (r*Fh*-GST-si); *F. hepatica* cathepsin L (r*Fh*-CL1)	Mouse DC and HEK293 cells	r*Fh*-GST-si, (but not r*Fh*-CL1) increased p65 phosphorylation and NF-κB activation.	Induction of IL-6, IL-12p40, and MIP-2, but also prevented IL-17 and IL-23 release.
*Fasciola hepatica* [150]	*F. hepatica* cathepsin L (*Fh*-CL3)	Mouse bone marrow-derived DCs	No change in p65 expression and IκB-α.	Increased release of IL-1β and IL-18 by NLRP3 dependent mechanism.
*Schistosoma mansoni* [151]	Infected mice	Liver cells	Increase in p65 expression and translocation to nucleus.	Possible role of p65 in inhibiting apoptosis.

## Data Availability

No new data were collected or analyzed in this study.
